# Structural and dynamic properties of IL1 receptors

**DOI:** 10.3389/fimmu.2025.1714624

**Published:** 2025-12-03

**Authors:** J. V. Zhukova, J. A. Lopatnikova, S. V. Sennikov

**Affiliations:** Laboratory of Molecular Immunology, Federal State Budgetary Scientific Institution “Research Institute of Fundamental and Clinical Immunology” (RIFCI), Novosibirsk, Russia

**Keywords:** IL-1 receptors, molecular mechanisms, interdomain flexibility, conformational dynamics, multi-domain proteins

## Abstract

The study of the structural and dynamic properties of multidomain proteins is of great interest as they constitute one of the main regulatory mechanisms in the ligand–receptor system. The interleukin-1 (IL-1) family is a group of proinflammatory, pleiotropic cytokines whose activity is primarily mediated through two primary signaling receptors—the IL-1 type I receptor (IL-1R1) and the IL-1 receptor accessory protein (IL-1R3), which acts as a coreceptor—and a decoy receptor, the IL-1 type II receptor (IL-1R2). Their key ligands include the agonists IL-1α and IL-1β, and the endogenous antagonist IL-1 receptor antagonist (IL-1Ra). Evaluating the interdomain movements of these receptors is expected to shed light on the mechanisms of ligand binding and the development of a functional cellular response. Furthermore, analysis of the literature allows us to identify key aspects of the conformational dynamics of IL-1 receptors. It is established that the flexibility of the domains of these receptors plays a crucial role in their ability to adapt to various ligands and ensure accurate signal transmission in the cell. Analysis of the structural and functional features of these receptors allows us to determine the mechanisms underlying their interactions and identify differences in their functional activities. Understanding the structural mechanisms that give rise to domain flexibility can enable us to develop molecules which are capable of specifically modulating the activity of these receptors. This facet is especially important in the context of treating inflammatory and autoimmune diseases for the creation of new approaches to therapy.

## 1 Introduction

Scientific interest in the molecular mechanisms that regulate the immune response is constantly growing (1). The molecular components of immunity include many examples of “multidomain proteins”. Yet, despite their ubiquity and critical functional roles, these proteins’ structural and dynamic properties remain poorly characterized (2, 3). A precise, quantitative understanding of interdomain motion is essential for elucidating the molecular recognition mechanisms of such proteins (4, 5). Recent studies have shown that the diversity of sequences and conformational flexibility of proteins induce universality in the regulation of cellular processes due to the formation of macromolecular structures (6–8). The IL-1 system is a critical regulator of inflammation and immune responses, and its signaling is tightly controlled. The three main receptors of this system play distinct roles: IL-1R1 is the primary signaling receptor, IL-1R3 is an essential co-receptor for signaling, and IL-1R2 is a “decoy” receptor that acts as a potent natural inhibitor (9).

IL-1 receptors belong to the Immunoglobulin Superfamily (Ig-SF). Members of this family share a common structural motif called the Ig domain, which is evolutionary ancient and used in a vast array of proteins involved in recognition, binding, and adhesion (10).

The main receptors in the IL-1 system (IL-1R1, IL-1R2, and IL-1R3) share a common extracellular architecture: three immunoglobulin-like (Ig) domains (D1, D2, and D3) (11), their functional outcomes (signaling *vs*. inhibition) are dictated by key structural differences and, crucially, their dynamic properties (10).

IL-1 receptors play a crucial role in pathology. The fundamental principle in this regard is that the disease often results from an imbalance between pro-inflammatory signaling and its natural inhibition. Excessive signaling (via IL-1R1/IL-1R3) drives pathology in auto-inflammatory, autoimmune, and metabolic diseases. Insufficient inhibition (via IL-1R2) cannot control inflammation, contributing to chronicity. Inappropriate decoy activity (via IL-1R2) can sometimes be exploited by pathogens or tumors to suppress beneficial immune responses (9).

IL-1R1 and IL-1R3 together form the functional receptor complex that transduces all the pro-inflammatory effects of IL-1β and IL-1α. Therefore, their overexpression or overactivation is directly pathogenic (11).

Autoinflammatory syndromes, characterized by innate immune dysregulation, recurrent fever, and systemic inflammation, are prime examples of IL-1β/IL-1R1 pathway hyperactivity, largely resulting from a failure to control IL-1-mediated signaling. The pathogenesis of monogenic autoinflammatory diseases directly involves the dysregulation of this pathway. For instance, gain-of-function mutations in the NLRP3 gene lead to uncontrolled inflammasome activation and excessive IL-1β production in cryopyrin-associated periodic syndromes (CAPS) (12). Conversely, loss-of-function mutations in *IL1RN*, the gene encoding IL-1Ra, cause deficiency of the IL-1 receptor antagonist (DIRA), leaving IL-1 signaling unopposed (13).

The most definitive proof of IL-1’s pivotal role is the profound clinical efficacy of targeted therapies. Drugs that block the IL-1 pathway, such as the recombinant IL-1Ra anakinra, the soluble decoy receptor rilonacept, and the anti-IL-1β monoclonal antibody canakinumab, induce rapid and sustained remission across a spectrum of diseases, from monogenic syndromes like CAPS and DIRA to polygenic conditions like systemic juvenile idiopathic arthritis and gout (14). This clinical success solidifies the IL-1 pathway as a critical therapeutic target in autoinflammation.

IL-1β is a key cytokine in Rheumatoid Arthritis (RA), driving synovitis, cartilage degradation, and bone erosion (15). IL-1R1 is considered an inflammation-related diagnostic biomarker for RA (16). Expression of IL-1R1 is upregulated on synovial fibroblasts and chondrocytes in the inflamed joint, making them hyper-responsive to IL-1 and thereby perpetuating destruction (17). The body employs several natural mechanisms to regulate this inflammatory signal. The IL-1 Receptor Antagonist (IL-1Ra) competes with IL-1 for binding to IL-1R1, thereby blocking its activation. Furthermore, a decoy receptor, IL-1R2, can bind IL-1β and prevent it from interacting with IL-1R1. This decoy receptor can also be released in a soluble form to neutralize IL-1β in the extracellular environment (18). In RA regulatory balance is disrupted. Research shows that the expression of membrane-bound receptors is altered on immunocompetent cells. For instance, monocytes from RA patients exhibit a decreased percentage of IL-1R1-positive cells but a significant increase in the density of IL-1R1 receptors on their surface, which may heighten their responsiveness to the cytokine. The balance between the signaling IL-1R1 and the decoy IL-1R2 is also disturbed, favoring a pro-inflammatory state. These changes in receptor expression represent a dynamic mechanism that modulates the cellular response to IL-1β in RA (19) The critical role of this axis is underscored by the therapeutic use of IL-1-blocking agents, such as the recombinant IL-1Ra Anakinra, which can reduce inflammation (20). The complex interplay between IL-1 cytokines, their signaling and decoy receptors, and natural antagonists is therefore a fundamental component of the chronic inflammation that characterizes Rheumatoid Arthritis.

In metabolic disorders, IL-1β (produced in response to forms of metabolic stress like high glucose levels) acts on pancreatic β-cells via IL-1R1 to impair function and induce apoptosis (21). In blood vessels, IL-1 signaling promotes atherosclerosis by activating endothelial cells and driving plaque inflammation (22).

IL-1R1’s role in cancer is complex and context-dependent, but it is increasingly recognized as a key driver of tumor progression, metastasis, and immunosuppression (23). Chronic inflammation is a well-known enabling characteristic of cancer, and IL-1 signaling, primarily through IL-1R1, is a primary regulator of the inflammatory response within the tumor microenvironment (TME), where its activation promotes a vicious cycle of tumor growth and spread (24). A key mechanism by which IL-1R1 drives cancer progression is by orchestrating an immunosuppressive TME. Signaling through IL-1R1 on stromal and immune cells stimulates the production of factors that recruit myeloid-derived suppressor cells (MDSCs), which potently suppress the activity of cancer-killing T cells and Natural Killer (NK) cells (25). Furthermore, IL-1 signaling promotes the differentiation of monocytes into M2-like macrophages, which are not anti-tumor; instead, they promote tissue repair, angiogenesis, and immunosuppression, all of which benefit the tumor (26). Additionally, IL-1β can impair the function of dendritic cells (DCs), preventing them from effectively presenting tumor antigens and activating T cells (27).

IL-1R2 is a decoy receptor that negatively regulates IL-1 signaling by binding IL-1α and IL-1β without initiating signal transduction. It exists in both membrane-bound and soluble forms, which sequester ligands and their accessory proteins. Expressed on neutrophils and monocytes, IL-1R2 is modulated by anti-inflammatory stimuli and plays a critical role in controlling inflammation in infections, autoimmune diseases, and cancer. Its soluble form is a potential biomarker for various inflammatory conditions (28).

The membrane-bound expression of IL-1R2 on monocytes serves as a key severity biomarker in sepsis. In septic patients, this decoy receptor is upregulated on circulating monocytes and correlates with the ensuing cytokine storm and immune dysfunction. Interestingly, its expression is highest in sepsis but declines in septic shock, where this lower level becomes a prognostic marker for mortality (29).

This dynamic expression is linked to a distinct cellular process. During severe infections, a unique population of transitional cells emerges in the circulation. These are not typical monocytes but have begun differentiating into macrophages while still in the blood, a state defined by a specific “monocyte-to-macrophage differentiation signature.” IL-1R2 is a key component of this signature; its expression is strongly upregulated as the cells acquire macrophage-like features. Therefore, the presence of IL-1R2 on a monocyte identifies it as part of this dysregulated population, directly linking the receptor to the severity of the inflammatory response (29).

In Inflammatory Bowel Disease, a relative deficiency in IL-1R2 expression or function in the gut mucosa may lead to a failure to adequately counterbalance local IL-1 production, perpetuating chronic inflammation (30).

In cancer, IL-1R2 can promote a state of immune tolerance by dampening IL-1 signaling (31). This role is exemplified in regulatory T cells (Tregs), which single-cell RNA sequencing has identified as primary expressers of this decoy receptor. Tregs sequester available IL-1β in the tumor microenvironment (TME), a mechanism that positions IL-1R2 as an active orchestrator of tumor immune escape (32).

The critical function of IL-1R2 extends beyond Tregs. For instance, a study in triple-negative breast cancer (TNBC) identified IL1R2 expression in a specific subset of tumor-associated macrophages (TAMs), where it acts as a master regulator of immunosuppression. Its primary function is to sequester IL-1β, preventing it from signaling. The study demonstrated that blockade of IL1R2 significantly attenuated macrophage recruitment, TAM polarization, and CD8+ T cell depletion. This intervention resulted in a reduced tumor burden and increased survival in mouse models, underscoring its therapeutic relevance (33).

Furthermore, the prognostic significance of IL-1R2 is evident in other cancers. An activated Treg signature, which includes IL-1R2 expression, as well as the IL1R2 gene itself, have been identified as independent prognostic markers associated with poor survival in lung adenocarcinoma (34).

## 2 Structural biology of the extracellular domain of IL-1 receptors

The structural biology of the Interleukin-1 (IL-1) receptor system, elucidated primarily through the crystal structures of its complexes with an antagonist and an agonist, revealed a novel binding mode distinct from other known cytokine-receptor families. This architecture provides an elegant molecular explanation for how a single receptor can distinguish between agonists that activate signaling and a natural antagonist that blocks it.

### 2.1 Overall structure

**Figure 1:**
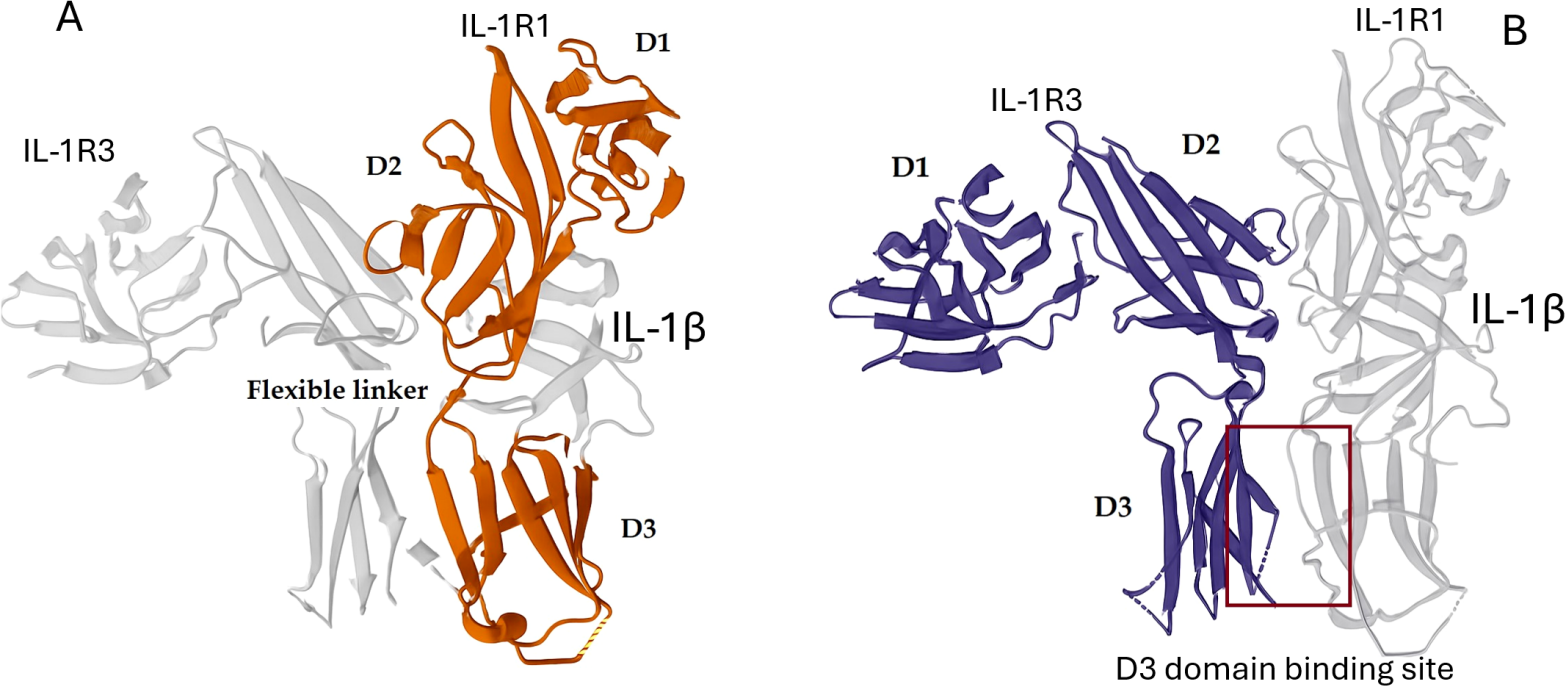
Structural architecture of the IL-1R1 and IL-1R3 signaling complex, with IL-1R1 and IL-1R3 highlighted in color. **(A)** Structural architecture of IL-1R1. The three Ig-like domains (D1, D2, D3) of IL-1R1 wrap around the ligand in a curved conformation. The flexible linker between domains D2 and D3 is highlighted. **(B)** Structural architecture of IL-1R3. This panel shows the interface between the D3 domain of IL-1R1 and the co-receptor IL-1R3. This interaction is critical for forming the high-affinity signaling complex. Protein structures were derived from PDB ID 4DEP (https://doi.org/10.2210/pdb4dep/pdb). Primary publication: https://doi.org/10.1038/nsmb.2260.

The extracellular ligand-binding portion of the type I IL-1 receptor (IL-1R1) is composed of three immunoglobulin (Ig)-like domains (35, 36). These domains are arranged in a distinctive curved shape that wraps around its ligand, resembling a “question mark” (36) (**Figure 1**).

Domains 1 and 2 are tightly associated, forming a rigid, integrated module. A conserved disulfide bond helps stabilize this interface (35). Domain 3 is connected to Domain 2 by a long, flexible linker, allowing it significant conformational freedom (36) (**Figure 1**).

This three-domain architecture is central to the IL-1 receptor’s unique function.

Unlike other cytokine-receptor complexes such as growth factor (37) and interferon-γ (38), where the ligand is sandwiched between two receptor molecules, the IL-1 system operates differently. In the IL-1 complex, a single receptor molecule uses its curved structure to engage the ligand, which sits nestled in the cavity formed by all three domains (35). This represented a paradigm for cytokine recognition.

The molecular architecture of IL-1β has been precisely defined through high-resolution techniques like X-ray crystallography and solution-state NMR. This cytokine adopts a highly conserved β-trefoil conformation, a stable and compact fold characterized by a central hydrophobic core built from twelve anti-parallel β-strands. Within this structure, six of these strands—specifically β1, β4, β5, β8, β9, and β12—assemble into an anti-parallel β-barrel, a key structural element. The overall β-trefoil fold is itself composed of six repeating β-hairpin motifs, which come together to form this durable and evolutionarily conserved three-dimensional scaffold (39–41).

Structural studies revealed that IL-1β engages the receptor through two distinct binding sites, which are the key to understanding signal regulation.

Site A (The Common Site). This site is on the “side” of the IL-1β β-barrel and is composed of residues that are conserved across all three natural IL-1 ligands (IL-1α, IL-1β, and IL-1ra). Site A makes extensive contacts with Domains 1 and 2 of the receptors (35). Mutagenesis studies confirmed that residues in this region are critical for binding all ligands (42).

Site B (The Agonist-Specific Trigger Site): This site is located on the “top” of the IL-1β β-barrel. Crucially, this site is only present and properly configured in the agonists IL-1α and IL-1β and is absent in the antagonist IL-1ra (35). Site B interacts exclusively with the Domain 3 (43).

### 2.2 The molecular mechanism of antagonism *vs*. agonism

**Figure 2:**
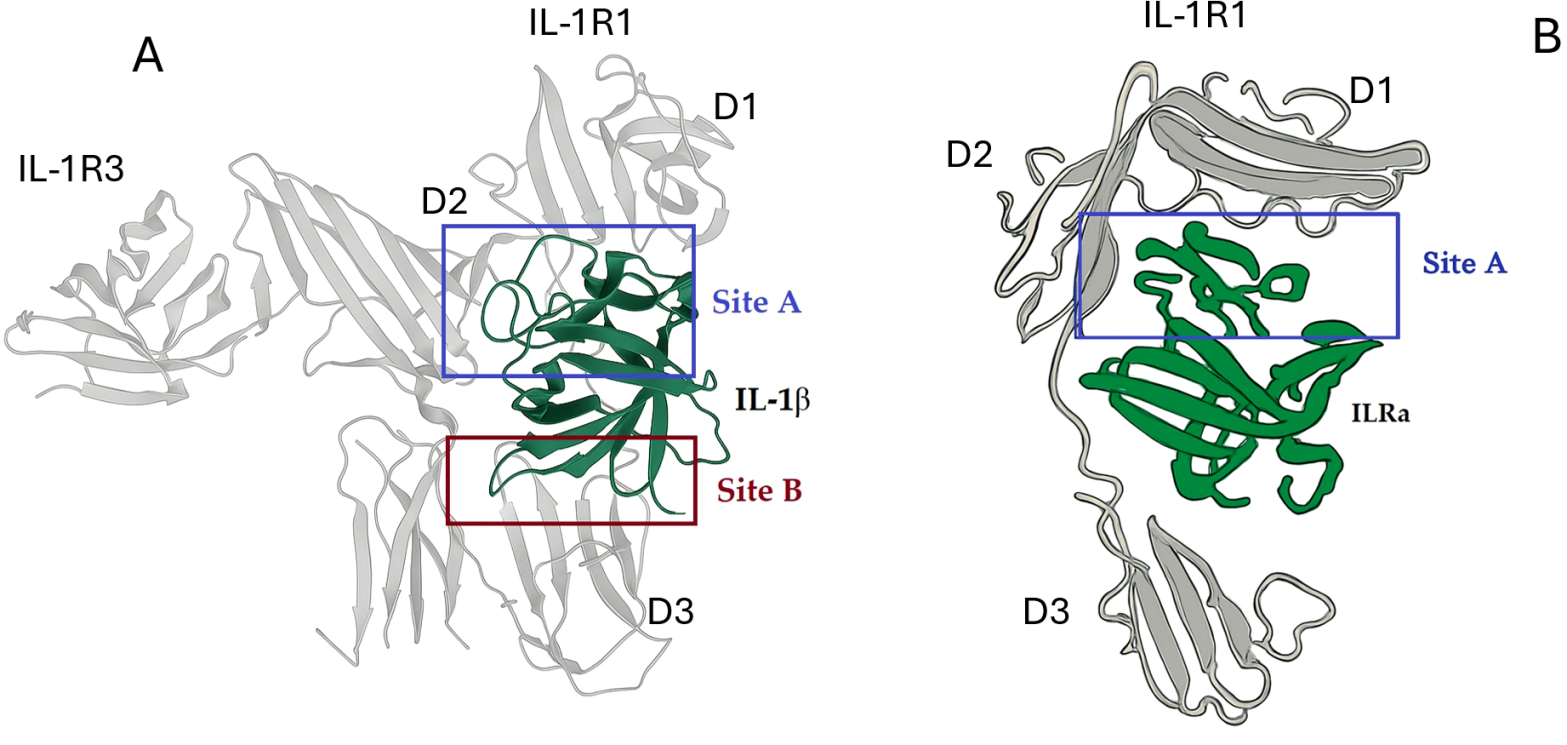
Structures of IL-1β and IL-1Ra and their interfaces with IL-1R1. **(A)** Overall structure of IL-1β in complex with the D1, D2, and D3 domains of IL-1R1. The binding interface is composed of two sites: Site A (IL-1β and the D1-D2 module) and Site B (IL-1β and the D3 domain). **(B)** Overall structure of IL-1Ra in complex with the D1 and D2 domains of IL-1R1. IL-1Ra binds to Site A but does not engage Site B, preventing the formation of the active signaling complex. Protein structures were derived from the following PDB entries: **(A)** 4DEP (https://doi.org/10.2210/pdb4dep/pdb) [Primary publication: https://doi.org/10.1038/nsmb.2260] **(B)** 1IRA (https://doi.org/10.2210/pdb1ira/pdb) [Primary publication: https://doi.org/10.1038/386194a0].

The structural data provides a model for how the receptor differentiates its ligands:

Antagonist Binding (IL-1Ra): IL-1ra binds with high affinity to Domains 1 and 2 via Site A (**Figure 2**). However, due to key structural differences in its loops (specifically the β4-β5 and β11-β12 loops) (44), it does not engage Domain 3 effectively. The receptor remains in an “open” conformation, unable to proceed to the next step of signal transduction (36) (45),. The failure to bind Site B means the D3 domain of IL-1RI remains in a different, “open” position. The functional state (agonist *vs*. antagonist) is not solely determined by the receptor-binding interfaces but can be allosterically controlled by a distant region of the protein. A specific mutation switches IL-1Ra from an antagonist to an agonist. The mutant protein can now activate NF-κB signaling in cells, just like IL-1β. Hydrogen-Deuterium Exchange Mass Spectrometry (DXMS) showed that when the mutant binds to IL-1RI, it causes destabilization in specific β-strands, effectively “unlocking” the protein. This allows the mutant IL-1Ra to now engage both receptor binding sites (A and B), making it signaling-competent and enabling it to recruit IL-1R3 (46).

Agonist Binding (IL-1β): The agonist binds tightly to Domains 1 and 2 via Site A. Simultaneously, its Site B engages Domain 3. (**Figure 2**) It was proposed that this dual engagement pulls Domain 3 downward, inducing a “closed” conformation of the receptor (36). This conformational change is the critical switch that creates a new surface, enabling the recruitment of the essential second receptor subunit, the L-1 Receptor Accessory Protein (IL-1R3), to form the active signaling complex (47). This model was strongly supported by functional studies. A truncated IL-1R1 containing only Domains 1 and 2 bound IL-1ra with high affinity but lost high-affinity binding to IL-1β, proving that Domain 3 is essential for agonist, but not antagonist, function (36) (48).

### 2.3 The ternary signaling complex

Subsequent research showed that the agonist-bound, closed conformation of IL-1R1 presents a perfectly complementary surface for IL-1R3 binding.

The assembly and activation of the interleukin-1β (IL-1β) signaling complex represents a fundamental process in inflammatory immunity. Through a series of complementary structural biology studies, the molecular mechanism governing this process has been elucidated. The foundational work provided the first atomic-resolution view of any IL-1 family ternary complex, revealing the structure of IL-1β in complex with the decoy receptor IL-1R2 and the signaling co-receptor IL-1R3. This structure established several critical principles. It demonstrated that IL-1R3 engages the pre-formed IL-1β–primary receptor complex in a “LEFT-side” architecture, binding to a composite surface created by both the cytokine and the receptor’s membrane-proximal domains (D2 and D3). The N-terminal domain (D1) of IL-1R3 projects away from the complex and is functionally dispensable for binding (44).

The subsequent study determined the crystal structure of the bona fide signaling complex, comprising IL-1β, its signaling receptor IL-1R1, and IL-1R3. This work confirmed that the “LEFT-side” architecture is conserved in the active, signal-competent complex. The structure showed that the binding of IL-1R3 induces only minor conformational changes in the pre-formed IL-1β–IL-1R1 binary complex. The resulting heterotrimeric assembly brings the D3 domains of IL-1R1 and IL-1R3 into proximity, thereby positioning their intracellular Toll/IL-1 receptor (TIR) domains to initiate downstream signaling cascades (49).

The most detailed mechanistic insights emerged from the comprehensive study which employed a multi-faceted approach including X-ray crystallography, hydrogen-deuterium exchange mass spectrometry (HDX-MS), surface plasmon resonance (SPR), and alanine-scanning mutagenesis. This research confirmed that IL-1R3 utilizes a conserved set of interaction motifs to engage different cytokine-receptor pairs. These include a central hydrophobic patch around IL-1R3, a cytokine-sensing loop that contacts both the ligand and the primary receptor, and polar interactions involving the D2-D3 linker region of IL-1R3. The energetic analysis revealed that IL-1β itself contributes nearly half of the binding energy for IL-1R3 recruitment, with key hotspots located on its β4–β5 and β11–β12 loops. This underscores a mechanism where the primary receptor IL-1RI acts primarily to present the cytokine in the correct orientation for high-affinity engagement with the shared co-receptor (50).

### 2.4 Decoy receptor

The Type II IL-1 Receptor, or IL-1R2, serves as the archetypal example of elegant form of regulation (51).

The extracellular portion of IL-1R2 is composed of three immunoglobulin-like domains, an architecture virtually identical to that of its signaling counterpart, IL-1R1. This structural mimicry allows IL-1R2 to bind the inflammatory cytokine IL-1β with an affinity comparable to the true receptor. However, a critical difference lies in its intracellular structure: IL-1R2 possesses only a short, truncated cytoplasmic tail (54). This deficiency renders it completely incapable of signal transduction, defining its primary role as a decoy.

The mechanism of inhibition is multifaceted and potent. Primarily, IL-1R2 acts through simple yet effective ligand sequestration. By binding IL-1β, it physically prevents the cytokine from engaging the functional IL-1R1, thereby directly neutralizing its inflammatory potential (52). Furthermore, the IL-1β/IL-1R2 complex can recruit the essential co-receptor, IL-1R3, which is normally required to form an active signaling complex with IL-1R1 (53). The resulting stable, three-part complex (IL-1β/IL-1R2/IL-1R3) is utterly inert. This process not only neutralizes the cytokine but also actively sequesters a key component of the signaling machinery, providing a second layer of inhibition (29).

The inhibitory reach of this system is further amplified systemically. The extracellular domain of IL-1R2 can be cleaved to form a soluble version (sIL-1R2) that circulates throughout the body. This soluble decoy acts as a scavenger, “mopping up” excess IL-1β in blood and tissues. The formation of a soluble ternary complex with the soluble co-receptor (sIL-1R3) creates natural inhibitors of IL-1 in the body (29).

Role of IL-1R2 is so crucial that it is now considered a key immune checkpoint, with its dysfunction linked to a variety of inflammatory diseases and pathological conditions (54).

## 3 Conformational dynamics of IL-1 receptors

To accurately understand the distinct properties and functions inherent in all natural molecules, one must first observe their structure and then their dynamic behavior over time. The study of molecular dynamics provides critical insights into how molecules function and move through space, with the specific experimental techniques applied varying according to the unique physicochemical properties of the target molecule and the desired spatial and temporal resolution. The goal of observing and predicting these dynamics is to gain a precise understanding of the underlying chemical and physical mechanisms. This knowledge not only advances academic understanding but also holds significant potential for practical applications in fields like medicine.

Accelerated molecular dynamics simulations were employed, which efficiently revealed the full scope of the D3 domain’s motion. These simulations showed the D3 domain undergoes a wide rotational sweep, with longitudinal and latitudinal motions spanning about 110 and 100 degrees, respectively. This extensive sampling led to the discovery of novel IL-1R1 conformations not previously observed in crystal structures. Analysis of these simulated conformations identified transient, potential small molecule binding sites. Three consensus locations emerged: the P1 site, situated between the D1 and D2 domains; the P2 site, located at the flexible D2-D3 junction; and the P3 site, found at the interface between D1 and D3 in inactive-like conformations. Among these, the P2 site was characterized as a promising allosteric modulator site. Its potential as a binding hotspot was further confirmed through mapping simulations (55).

Small-Angle X-Ray Scattering (SAXS) show the ectodomains of IL-1 receptors fluctuating between “open” and “closed” states. The signaling receptor IL-1R1 is highly flexible, while the decoy receptor IL-1R2 exhibits a strikingly different dynamic profile, preferring a more rigid, predominantly closed conformation. Molecular Dynamics (MD) simulations provide an atomic-resolution view of these motions, identifying the D2/D3 linker as the epicenter of this flexibility. In IL-1R1, specific residues like Glu202, Glu203, and Asn204 act as a central pivot—the “central wheel of a clock’s movement”—enabling significant hinge-bending and rotational motions of the D3 domain relative to the more rigid D1-D2 module (56).

The functional necessity of this precisely tuned dynamic repertoire is starkly demonstrated by experimental studies. When the natural D2/D3 linker sequence in the essential co-receptor IL-1R3 is replaced with linkers from other receptors, including the one from IL-1R2, its biological function is completely abolished. The mutated co-receptor loses all ability to bind the pre-formed IL-1/IL-1R1 complex and fails to initiate downstream NF-κB signaling. This failure is not due to global misfolding, disrupted binding interfaces, or mislocalization, but rather to a fundamental alteration in its conformational landscape. SAXS analyses confirm that the linker-swapped mutants, such as IL-1R3 with the IL-1R2 linker, explore a different and more restricted set of conformations. The natural linker sequence is therefore a critical functional determinant, encoding the specific dynamic repertoire that allows the co-receptor to sample the “binding-competent” state necessary for productive complex assembly (56).

The distinct conformational behavior of IL-1R2 itself is directly linked to its biological role as a decoy. The closed conformation is perfectly suited for its function: it can bind and sequester IL-1 cytokines with high affinity but, due to lack of a TIR domain, it cannot recruit the co-receptor into a productive signaling complex. Instead, it can form a non-signaling ternary complex with IL-1R3, further dampening the inflammatory response. This illustrates how nature has harnessed the same structural fold—three Ig-like domains and a D2/D3 linker—but, through sequence variation, has tuned their conformational dynamics to achieve opposing functions: activation for IL-1R1 and inhibition for IL-1R2 (56).

The soluble IL-1R1 demonstrated remarkable flexibility, undergoing large-scale “open-to-closed” transitions and samplingx“twisted” conformations where the D3 domain rotates significantly. This suggests a high degree of structural plasticity in solutions. Conversely, the membrane-bound domain was more conformationally restrained, sampling a narrower range of states and exhibiting fewer extreme transitions. This restriction is attributed to the physical presence of the membrane, which acts as a steric hindrance and likely imposes specific orientational constraints on the extracellular domain, potentially pre-organizing it for signaling (57).

The 6-amino-acid linker between domains D2 and D3 was confirmed as the central structural element governing domain orientation. A detailed dihedral angle analysis identified residues Glu202, Glu203, and Asn204 as the primary source of flexibility, with their backbone torsion angles showing the largest variations during conformational changes. The study metaphorically describes this region as the “central wheel of a clock’s movement,” driving the reorientation of the ligand-binding D3 domain (57).

Quantitative analysis revealed that closed conformations, characterized by closer proximity between D1 and D3, were associated with a higher number of intramolecular hydrogen bonds, suggesting they may represent more stable, low-energy states. The soluble form was able to access a wider diversity of these closed and twisted-closed states compared to the membrane-bound form (57). 

These computational findings have significant biological implications. The heightened flexibility of the soluble IL-1R1 may be crucial for its role as a decoy receptor, enabling it to trap a diverse array of IL-1 ligands in the circulation with high avidity. The more restricted dynamics of the membrane-bound form, however, may reflect a functional requirement for a specific conformational state that is optimally primed for the recruitment of the essential co-receptor, IL-1R3, upon ligand binding, thereby ensuring precise signal initiation. The process begins with the ligand-induced conformational stabilization of IL-1R1. In its unbound state, the three immunoglobulin (Ig) domains of IL-1RI are flexible. Upon binding IL-1β, the receptor undergoes a large-scale conformational change, wrapping its domains around the cytokine in a characteristic “grasping hand” or “question mark” architecture. This forms a stable IL-1β/IL-1R1 binary complex that serves as a rigid platform. Crucially, the structure of this binary complex remains virtually unchanged upon subsequent binding of IL-1R3, indicating its role as a pre-formed scaffold (49) (58),. This dual engagement is thought to stabilize an active conformation of the IL-1R1 complex, facilitating the association of IL-1R3 (59). This binding event is not passive; the ligand actively selects and stabilizes a specific, active conformation from the receptor’s natural range of motion, often involving a dramatic reorientation of its domains.

In stark contrast, the binding of the antagonist IL-1Ra results in an abortive complex. The central question addressed in studies is the conformational nature of this trimeric, signal-competent complex. Through molecular modeling, two distinct modes of interaction were predicted. In the FRONT model, IL-1R3 docks directly onto the primary IL-1β/IL-1R1 interface. Conversely, the BACK model positions IL-1R3 against the “back” of IL-1R1, specifically interacting with its third domain while also contacting adjacent regions on IL-1β itself. Experimental evidence strongly supports the BACK model as the activating conformation. Critical data come from IL-1β mutants that retain full ability to bind IL-1R1 but exhibit a profound loss of biological activity. Computational analysis of these mutant residues shows that their positions are significantly closer to the predicted IL-1R3 interface in the BACK model than in the FRONT model (60). More recent studies have shown that the trimeric complex has a much thinner profile than the ‘front’ or the ‘back’ models. This binding event is not passive; the ligand actively selects and stabilizes a specific, active conformation from the receptor’s natural range of motion (61). Currently, the LEFT architecture is final, based on the crystal structure (44).

Conformational dynamics of the IL-1 system illustrate a precise molecular switch. The transition from a binary, inactive complex to a trimeric, active one is governed by the specific wrap-around conformation induced by agonist binding, which unveils a cryptic site for IL-1R3 docking (**Table 1**).

**Table 1:** Comparison of the interdomain flexibility of IL-1 receptors.

Receptor	Type	Relative flexibility	Key evidence and characteristics
IL-1R1	Primary Receptor	1 (Most Flexible)	• SAXS: Exhibited the most flexible normalized Kratky plot and a wide range of conformations in its ensemble. • MD: Sampled a wide conformational space, visiting open, closed, and “twisted” states. Its soluble form was more flexible than its membrane-bound form.
IL-1R3	Co-receptor	2 (Intermediate/Less Flexible)	• SAXS: Less flexible than IL-1R1 and ST2. Existed largely in a preferred “open” state. • Functional Data: Its natural D2/D3 linker sequence is critical. Replacing it abolished ternary complex formation and signaling by perturbing its conformational ensemble, not by disrupting direct binding contacts.
IL-1R2	Primary Receptor (Decoy)	3 (Least Flexible)	• SAXS: Identified as the least flexible receptor via Kratky plot. Its conformational ensemble was predominantly in a single, “closed” state. • This more rigid, pre-closed state is consistent with its role as a decoy receptor, potentially pre-formed to bind ligand but does not permit productive signaling.

Thus, it has been shown that interdomain flexibility of IL-1 receptors change their conformations for optimal binding to ligands. This property ensures they can effectively interact with various molecules, a critical capacity for their biological functions.

## 4 Structural architecture and functions of the IL-1 cytoplasmic domain

The TIR (Toll/Interleukin-1 Receptor) domain is a conserved protein interaction module of approximately 160 amino acids, located in the cytoplasmic tail of receptors and adaptors in the innate immune system. Its primary function is to nucleate the assembly of large signaling complexes through specific homotypic interactions, a process that is entirely dependent on its precise three-dimensional structure (62).

The structures of many TIR domains are generally similar. However, it has been extremely difficult to obtain stable TIR/TIR complexes in solution for structural studies. The TIR domain adopts a characteristic α/β fold, comprising a central, parallel five-stranded β-sheet (βA–βE) surrounded by five α-helices (αA–αE) on both sides. This compact structure is highly conserved and serves as a stable scaffold upon which variable loops and surfaces are displayed for specific binding events (63).

**Figure 3:**
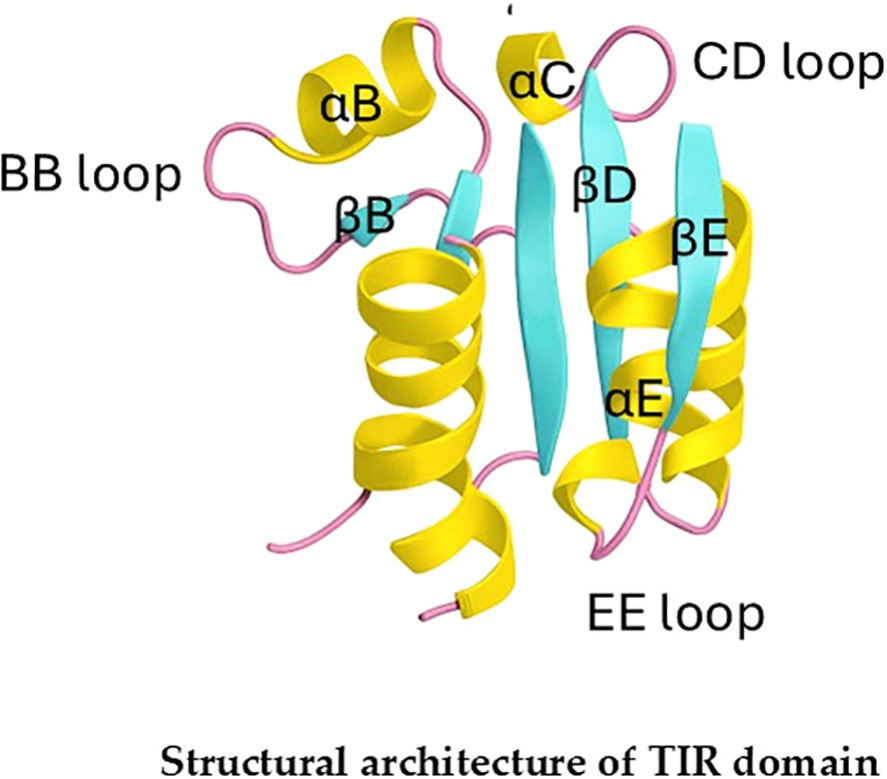
Schematic architecture of the TIR domain. The structure is based on the crystal structure of the human IL-1RAPL TIR domain. Key surface regions that mediate critical protein-protein interactions for downstream signaling are highlighted. The BB, CD, and EE loops are indicated. The protein structure was derived from PDB ID 1T3G (https://doi.org/10.2210/pdb1T3G/pdb). The primary publication is https://doi.org/10.1074/jbc.M403434200.

Our structural knowledge of the IL-1R TIR domain is extremely limited. The crystal structure of the IL-1RAPL TIR domain revealed similar structures and signaling mechanisms (**Figure 3**). Moreover, crystal structures of the TIR domains from IL-1R3b, IL-18Rβ, IL-1RAPL2, and SIGIRR (including its mutant SIGIRR-C299S) have revealed the presence of key functional segments mediating NF-κB activation. However, the relatively low sequence identity, in the range of 25–37% (except for 74% between IL-1RAPL1-TIR and IL-1RAPL2-TIR), indicates important differences that determine their specificity (64, 65).

Research on different types of TIR domains over the years has identified several key surface regions that mediate critical protein-protein interactions.

### 4.1 The BB loop and the αB helix (the canonical dimerization interface)

This is the most prominent and well-characterized functional surface. The BB loop, a protruding and flexible segment connecting the βB strand and the αB helix, forms a primary interaction patch together with the adjacent αB helix. This surface is essential for the heterodimeric interaction between the TIR domains of the signaling receptor (IL-1R1) and its essential accessory protein (IL-1R3) (66). Supporting this, the crystal structure of the IL-1RAPL TIR domain revealed that its BB loop and αB helix form a prominent, exposed surface primed for protein interactions (63). More recently, the cryo-EM structure of the active IL-1R1/IL-1R3 complex provided direct visual proof, identifying this interface as a central contact point between the two receptor TIR domains (65) (**Figure 3**).

### 4.2 The CD loop and β-strands (secondary stabilization interface)

Beyond the canonical interface, other regions contribute to the interaction network. The loop connecting the βC strand and αC helix (the CD loop), along with the surfaces of the βC and βD strands, are implicated in forming secondary contact points that stabilize the TIR heterodimer. These regions likely contribute to the specificity and affinity of the interaction, ensuring the correct pairing of receptor components. This was confirmed by mapping specific residues from the CD loop and adjacent β-strands that participate in the heterodimeric interface, revealing a larger and more complex interaction area than previously appreciated (65).

### 4.3 The EE loop (a potential regulatory interface)

Located on the opposite side of the domain from the BB loop, the EE loop comprises the region after the βE strand and the αE helix, and it may play a regulatory role. While its function in the core IL-1 receptor dimer is less defined than that of the BB loop, the EE loop has been implicated in TIR-TIR interactions in other systems. It therefore represents a potential surface for interactions with regulatory proteins (64, 67).

The binding of IL-1 to its receptors induces a conformational change that brings the intracellular Toll/Interleukin-1 Receptor (TIR) domains of the two receptor subunits into close proximity. This dimerized TIR domain platform then acts as a molecular beacon, recruiting the primary cytoplasmic adapter protein, Myeloid Differentiation Primary Response 88 (MyD88), through homotypic TIR-TIR interactions (68).

MyD88 is the central scaffold and orchestrating factor of the entire cascade. Its structure is key to its function: it possesses a C-terminal TIR domain that binds the receptor and an N-terminal Death Domain (DD) that drives the next critical step. The Death Domains of individual MyD88 molecules interact in a specific “head-to-tail” fashion, initiating the formation of a large, helical oligomer. This is not a random aggregation but a precisely controlled event that gives rise to the core signaling platform known as the Myddosome (69). The function of this oligomerization is twofold: it creates a stable platform for recruiting downstream kinases, and critically, the size of the MyD88 oligomer acts as a physical threshold. Only complexes exceeding a certain size can generate a signal strong enough to proceed, preventing accidental cellular activation (70). Recent evidence also shows that these individual Myddosomes can further cluster together, amplifying the signal to ensure robust activation of the downstream pathway (71).

**Figure 4:**
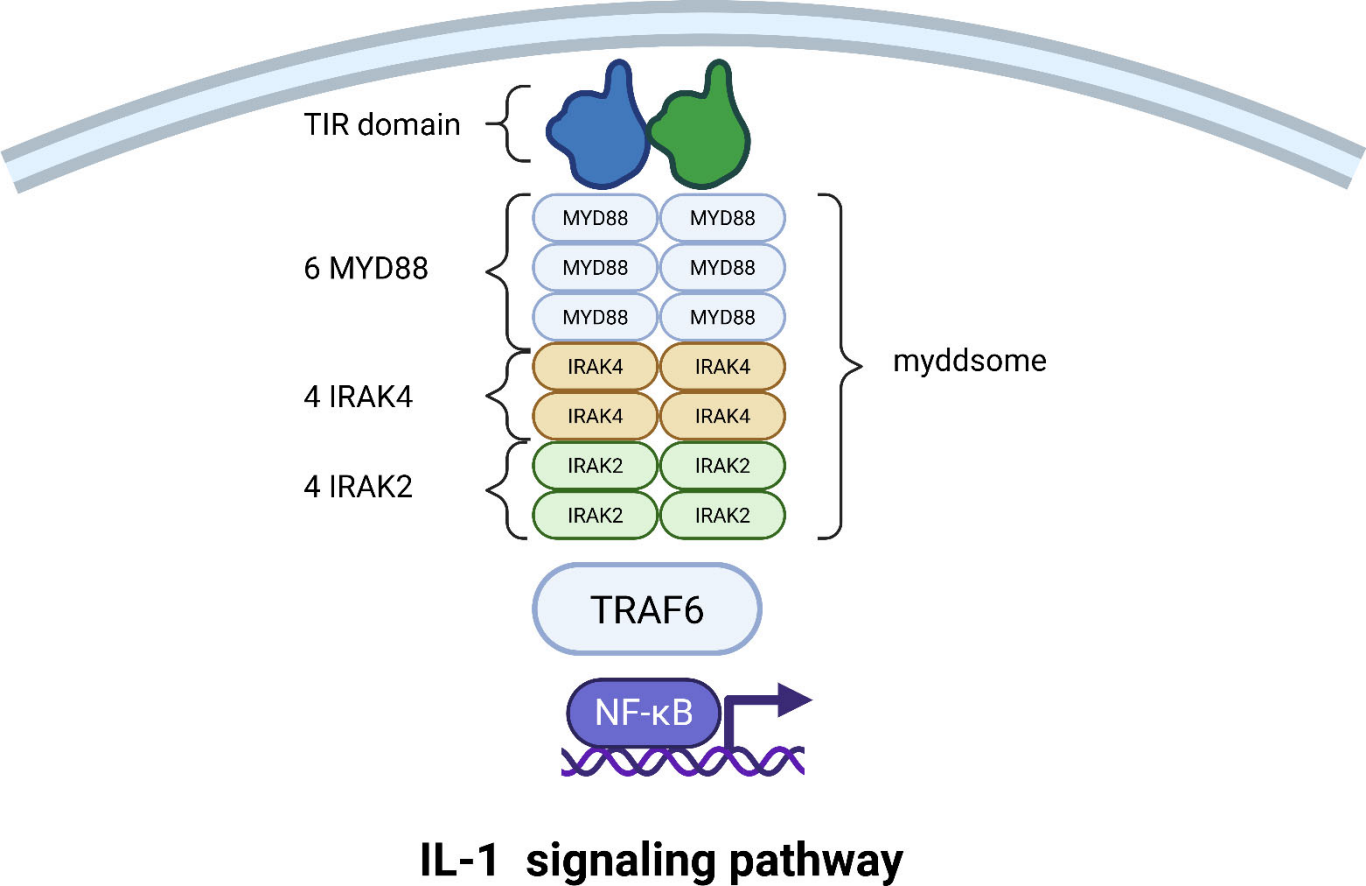
Assembly of the Myddosome and activation of the IL-1 signaling pathway. This schematic depicts the stepwise assembly of the core Myddosome signaling complex and the downstream events leading to NF-κB activation. Following the formation of the IL-1 receptor ternary complex, the adapter protein MyD88 is recruited, nucleating a helical oligomer. IRAK4 and IRAK2 kinases are then sequentially recruited to the complex through death domain (DD) interactions. Upon activation and release, IRAK2 recruits TRAF6, initiating a signaling cascade that culminates in the translocation of NF-κB to the nucleus and the induction of pro-inflammatory gene expression.

Into this helical MyD88 scaffold, the first kinase is recruited: Interleukin-1 Receptor-Associated Kinase 4 (IRAK4). IRAK4 is a serine/threonine kinase that also contains a Death Domain, allowing it to bind directly to the oligomeric Death Domain array of MyD88. Once incorporated, IRAK4 becomes activated through proximity-induced trans-autophosphorylation. The recruitment of IRAK4 then creates new binding sites for a second kinase, IRAK2. Like IRAK4, IRAK2 possesses a Death Domain that slots into the growing helical complex. This results in a stable Myddosome with a defined stoichiometry, often a 6:4:4 ratio of MyD88:IRAK4:IRAK2, forming a highly efficient kinase activation platform (72) (**Figure 4**).

The activated IRAK4 phosphorylates IRAK2 on specific activation loop residues, unleashing its full kinase activity. The phosphorylated IRAK2 then undergoes additional modifications (such as autophosphorylation) that reduce its affinity for the complex, causing it to dissociate from the stable Myddosome core. The IRAK kinases are thus pivotal signal transducers, and their recruitment, activation, and release are tightly regulated to control the immune response (73).

The liberated, activated IRAK2 now acts as a molecular bridge, recruiting Tumor Necrosis Factor Receptor-Associated Factor 6 (TRAF6). TRAF6 is a central regulator that changes the language of the signal from phosphorylation to ubiquitination. TRAF6 catalyzes the synthesis of long chains of ubiquitin linked through lysine 63 (K63). These K63-linked ubiquitin chains are then modified by a second complex called LUBAC (Linear Ubiquitin Chain Assembly Complex), which attaches ubiquitin molecules in a linear head-to-tail fashion through methionine 1 (M1), creating unique hybrid ubiquitin chains (74, 75).

These hybrid ubiquitin chains simultaneously recruit two critical kinase complexes. The first is the TAK1 (TGF-β-Activated Kinase 1) complex, composed of TAK1 (MAP3K7) and its regulatory subunits TAB1, TAB2, and TAB3. The second is the IKK (IκB Kinase) complex, which consists of the catalytic subunits IKKα and IKKβ and the essential regulatory scaffold NEMO (NF-κB Essential Modulator). The TAK1 complex is a central signal integrator in inflammation (76). Once recruited via NEMO’s affinity for the ubiquitin chains, the TAK1 complex is activated and phosphorylates the IKK complex, specifically on the activation loop of the IKKβ subunit. This activates the IKK complex, which then phosphorylates the inhibitor of κB, IκBα. IκBα normally binds to and sequesters the transcription factor NF-κB in the cytoplasm. Phosphorylation of IκBα marks it for rapid degradation, liberating NF-κB. NF-κB then translocates to the nucleus, where it binds to specific DNA sequences and initiates the transcription of inflammatory genes. Remarkably, the NF-κB system can decode the intensity of the initial signal, responding to absolute differences in cytokine concentration to ensure a proportionate cellular response (77) (**Figure 4**).

## 5 Therapeutic targeting of the IL-1 pathway

Understanding the conformational changes of IL-1 receptors is critical for developing new and effective therapeutic strategies for a wide range of inflammatory and immune-mediated diseases.

The interleukin-1 (IL-1) signaling pathway represents one of the most potent drivers of the inflammatory response in human physiology. The evolution from first-generation biologics to a current pipeline of sophisticated inhibitors exemplifies a broader shift in drug discovery—one increasingly guided by high-resolution structural and dynamic insights. This deep molecular understanding is now enabling a multi-pronged therapeutic assault on the pathway, moving beyond simple receptor blockade to allosteric modulation, co-receptor targeting, and even active immunization.

The initial therapeutic strategy was rooted in mimicking endogenous regulation using the natural IL-1 receptor antagonist (IL-1Ra), which occupies the IL-1R1 binding site without initiating signal transduction. Long-term clinical studies have established the real-world utility of this approach in conditions like Adult-Onset Still’s Disease (78). The variability in Anakinra’s efficacy was directly investigated in genetic studies, which found that polymorphisms in the *IL1RN* gene significantly affect treatment response in systemic juvenile idiopathic arthritis (79). This links a patient’s inherent genetic blueprint of the IL-1 system to therapeutic outcomes.

Building on this natural template, structural biology has enabled a rational design approach for creating superior antagonists. Recent research demonstrated this by computationally and experimentally optimizing the IL-1Ra structure, yielding a smaller, more stable protein with significantly higher binding affinity for IL-1R1 (80). Furthermore, fusing IL-1Ra to an Fc fragment creates a biotherapeutic with an extended serum half-life and enhanced efficacy in ameliorating autoimmune arthritis, showcasing how protein engineering can refine foundational biologic strategies (81).

The development of monoclonal antibodies against IL-1β highlighted that the precise location of binding—the epitope—is a critical determinant of therapeutic mechanism (82). Comparative structural analysis of Gevokizumab and Canakinumab revealed that although they neutralize IL-1β, they bind to distinct, non-overlapping epitopes. Canakinumab acts primarily through steric hindrance, physically blocking the cytokine’s access to IL-1R1, while Gevokizumab binds allosterically, inducing subtle conformational changes in IL-1β that reduce its binding affinity for the receptor without fully occluding the interface (82). This “one target, multiple binding modes” paradigm underscores that therapeutic efficacy is not merely a function of binding, but of the specific structural and dynamic consequences of that binding.

A major therapeutic advance came from looking beyond the primary ligand-receptor interaction. Signal transduction requires the recruitment of a co-receptor, IL-1 Receptor Accessory Protein (IL-1R3), to form a high-affinity ternary complex (83). This complex assembly presents multiple new vulnerabilities that can be exploited for more precise therapeutic intervention.

Research exploring antibodies against different epitopes on IL-1R3 demonstrated that the mechanism of inhibition is entirely epitope-dependent (83). Some antibodies prevent the initial recruitment of IL-1R3 to the IL-1/IL-1R1 binary complex, while others allow complex formation but disrupt the subsequent conformational change needed for intracellular signaling (83). This provides a toolkit for tailored intervention, allowing scientists to choose which specific step in the pathway to disrupt based on the desired therapeutic outcome.

At an even more granular level, characterization of specific critical residues on IL-1R3 has shown that disrupting single interaction points can be sufficient to block IL-1β signaling and exert protective effects in disease models (84). This approach proves the druggability of precise protein-protein interfaces within the complex and opens avenues for highly specific inhibitor development.

Beyond biologics, structural dynamics simulations have been instrumental in identifying targets for small-molecule drugs. For instance, computational methods have mapped the IL-1R1 ectodomain, predicting the existence of transient allosteric pockets that could be stabilized by small molecules to lock the receptor in an inactive state (85).

In parallel, the direct targeting of IL-1β itself with small molecules has been validated through comprehensive “ligandability assessment” using a combination of advanced biophysical techniques (86). These systematic approaches have proven that the surface of IL-1β contains druggable hotspots, with several chemical starting points identified that bind to these pockets and allosterically inhibit the IL-1β/IL-1R1 interaction, providing a solid foundation for lead optimization (86). Furthermore, this search for inhibitors is being accelerated by modern computational techniques (11). Machine learning approaches using quantitative structure-activity relationship modeling have enabled virtual screening for novel small-molecule inhibitors of the IL-1R1/IL-1β interaction, identifying promising new chemotypes with high predictive confidence (11).

The most revolutionary therapeutic perspective emerging from recent research is the concept of active immunization against IL-1 receptors (87). The preparation of these vaccines involves a sophisticated design to overcome the immune system’s natural tolerance to self-proteins. A common and effective method is virus-like particle (VLP) technology (87, 88). The process begins with the selection of a self-antigen, which can be the mature IL-1β cytokine or, in a more comprehensive newer strategy, the IL-1 Receptor Type I (IL-1R1) itself. This chosen antigen is then chemically conjugated or genetically fused to a carrier platform, specifically bacteriophage-derived VLPs. These VLPs are non-infectious as they lack viral genetic material, but their highly repetitive, particulate structure is exceptionally effective at triggering a strong immune response (88). The final vaccine is formulated from these VLP-cytokine or VLP-receptor conjugates in a standard saline buffer, often with the addition of approved aluminum-based adjuvants to further enhance the immune response upon injection (88).

The mechanism of action for these vaccines leverages the principles of adaptive immunity (89, 90). Following injection, the body’s antigen-presenting cells recognize the foreign, immunogenic VLP carrier. Because the self-antigen is physically attached to this “danger signal,” the immune cells engulf the entire construct and present its fragments to helper T cells. This process effectively breaks the natural immune tolerance, convincing the immune system that the self-cytokine or receptor is a foreign invader (88). This activation then prompts helper T cells to assist B cells in maturing and producing high-affinity, neutralizing antibodies specifically against the IL-1 target (90), thereby establishing a dedicated production line for the therapeutic agent within the patient. These vaccine-induced antibodies are released into the bloodstream, where they circulate throughout the body. They bind to and neutralize any circulating IL-1β cytokine or, in the case of receptor-targeting vaccines, bind to IL-1R1 on cell surfaces, physically blocking it from interacting with its natural ligands (87). This action prevents the IL-1 signal from being transduced to the nucleus, thereby shutting down the pro-inflammatory cascade that drives disease pathology (89).

The practical efficacy has been demonstrated in a model of type 2 diabetes, where an anti-IL-1β VLP vaccine successfully generated antibodies and improved metabolic function (88). A comprehensive review subsequently described the broad suitability of this strategy across various inflammatory conditions (90). The field continues to advance, with the most recent work focusing on novel vaccines that target the IL-1 receptor itself, offering a more potent and comprehensive blockade of the entire inflammatory pathway (87). In conclusion, anti-IL-1 vaccination represents a promising shift from chronic biologic treatment toward a potential one-time or occasional immunization, offering the prospect of durable suppression of inflammation through an elegantly engineered immune response.

## 6 Future directions and conclusions

Our analysis of the literature revealed key aspects of the conformational dynamics of IL-1 receptors. It is established that the flexibility of the domains of these receptors plays a critical role in their ability to adapt to various ligands and ensure accurate signal transmission in the cell. Analysis of the structural and functional features of the receptors has made it possible to determine the mechanisms underlying their interactions and identify differences in their functional activity.

Further study of the interdomain flexibility of receptors has significant potential to facilitate the development of new therapeutic approaches. Knowledge of the structural mechanisms that induce domain flexibility can be used to develop molecules that are capable of specifically modulating the activities of these receptors. This is especially important in the context of the treatment of inflammatory and autoimmune diseases. Studying the various mechanisms through which cytokine receptors are regulated is a promising endeavor. These studies are expected to help in deepening our understanding of the mechanics of their actions and open up new opportunities for therapeutic intervention.

## Glossary

CAPS: Cryopyrin-Associated Periodic Syndromes
DC: Dendritic Cell
DD: Death Domain
DIRA: Deficiency of the IL-1 Receptor Antagonist
HDX-MS: Hydrogen-Deuterium Exchange Mass Spectrometry
Ig-SF: Immunoglobulin Superfamily
IKK: IκB Kinase
IL-1: Interleukin-1
IL-1R1: Interleukin-1 Receptor Type I
IL-1R2: Interleukin-1 Receptor Type II (Decoy Receptor)
IL-1R3: Interleukin-1 Receptor Accessory Protein (Co-receptor)
IL-1Ra: Interleukin-1 Receptor Antagonist
IL-1RAcP: Interleukin-1 Receptor Accessory Protein (synonymous with IL-1R3)
IRAK: Interleukin-1 Receptor-Associated Kinase
K63: Lysine 63
LUBAC: Linear Ubiquitin Chain Assembly Complex
M1: Methionine 1
MD: Molecular Dynamics
MDSC: Myeloid-Derived Suppressor Cell
MyD88: Myeloid Differentiation Primary Response 88
NF-κB: Nuclear Factor Kappa-Light-Chain-Enhancer of Activated B cells
NK cell: Natural Killer cell
RA: Rheumatoid Arthritis
SAXS: Small-Angle X-Ray Scattering
sIL-1R2: Soluble IL-1 Receptor Type II
sIL-1R3: Soluble IL-1 Receptor Accessory Protein
SPR: Surface Plasmon Resonance
TAK1: TGF-β-Activated Kinase 1
TAB: TAK1-Binding Protein
TIR: Toll/Interleukin-1 Receptor
TME: Tumor Microenvironment
TNBC: Triple-Negative Breast Cancer
TRAF6: TNF Receptor-Associated Factor 6
Treg: Regulatory T Cell
VLP: Virus-Like Particle
